# An Elementary Treatise on Midwifery: Or Principles of Tokology and Embryology

**Published:** 1845-10

**Authors:** 


*An Elementary Treatise on Midwifery: or Principles of Tokology and Embryology.* By ALF. A. L. M. VELPEAU, M. D., &c. &c. Translated from the French by CHARLES D. MEIGS, M. D., member of the American Philosophical Society; Professor of Midwifery in the Jefferson Medical College, &c. &c. Third American edition, with notes and additions by WILLIAM HARRIS, M. D., Member of the American Philosophical Society; Lecturer on Midwifery and the Diseases of Women and Children, &c. &c. 8vo., pp. 600. Lindsay and Blakiston: Philadelphia, 1845.

Our readers must have been struck with the large number of treatises on Obstetrics, which we have had occasion to notice within the last year or two. Scarcely a work of merit has appeared in Great Britain or France without having been reprinted in this country: some of them, too, illustrated—really *illustrated*—by excellent lithographic plates, or wood engravings. Yet still there appeared to the respectable publishers, who have issued the work before us, room for an old favourite, which had been permitted to rest for a while, but had by no means been forgotten; and, accordingly, we have from them Velpeau *redivivus.* Our old friend appears, however, with a new face, and instead of having for its "gentleman usher and prolegomenon" the distinguished translator, teacher and obstetrician, Professor Meigs, we find it introduced by another. "As Professor Meigs, who translated the work, and enriched the second edition with a variety of valuable notes, is absent on a professional tour through Europe, the duties of bringing out a third edition have been assigned to me." So says the Editor; but we do not exactly comprehend the *sequitur.*

The work of Velpeau is one that has been highly prized by us for the extent of information it conveys, more especially on every point connected with obstetrical physiology. It is of course essentially French, and essentially French it must remain, in spite of the labours of all annotators: still it is an excellent addition to the library of the obstetrician, and a good guide on most points to the obstetrical student. The notes of Dr. Meigs are generally judicious: we wish we could say as much for those of his successor, which are often unnecessary or commonplace, at times merely confirmatory of what has been said in the text ; at others, neutralizing or positively opposing it ; and at others, again, consisting of assertions without adequate, or, indeed, any evidence being brought forward to confirm them. We have read them all over, and had the mortification to feel that our labour has been profitless; and to fear that such must be the case with those who are even less familiar with the subject than we are.

Amongst the "unnecessary" we would place the definitions of *tokology*, *coxal* bone, and *eutocia;* which, by the way, is stated to be from εν instead of ευ, well, and *Tokos*, childbirth, &c. &c.,—inasmuch as the terms are to be found in every medical dictionary. In describing the sacrum, M. Velpeau states, that along its middle are " four or five quadrangular facettes, and the same number of transverse lines." The Editor adds in a note " Five quadrangular facettes and four transverse seams." The sacrum, according to M. Velpeau, is still formed at birth of fifteen pieces, three for each vertebra. The Editor comments on the word " birth" as follows : " that is at the end of the seventh month of fœtal life." Several notes are added on the anatomy of the pelvis, of all points the one least requiring additions. M. Velpeau affirms, that "the axis of the superior strait is an imaginary line, extending from the umbilical region to the lower two-thirds of the front of the sacrum :"—not a very precise definition it is true. The Editor says it extends from the umbilicus to the junction of the sacrum and coccyx. At page 58, the labia are said to be sometimes enlarged by varicose veins ; and to be " also the seat of pruritus, of morpiones and of herpes,"—but itching may obviously occur in every part of the generative organs, whilst the insects referred to infest the mons veneris as well as the labia ; in other words all the parts of the sexual organs that are covered with hair. At page 64 we are gravely told, that the " introduction of a pessary generally destroys the hymen in *virgins*," and this is a note to a passage in the original, stating that "as a general rule the hymen is ruptured at the first sexual approach."

The perineum has hitherto been limited, posteriorly by the anterior margin of the anus—" *mais nous avons changé tout cela.*" It now, according to the Editor, extends to the os coccygis ! (p. 65.) At page 70, we are told that " it is now pretty clearly ascertained that the ovaries are the efficient cause of menstruation, and that one or more Graafian vesicles are ruptured at each menstrual period. This is often followed by a *corpus luteum,* and hence the presence of this *round yellowish body* is no longer a test of previous *gestation.*" This, however, is a foregone conclusion. The Editor will find, in the recent journals, that Raceborski, one of the great supporters of the modern doctrine of the agency of the ovaries in menstruation, has been vigorously engaged in examining into, and publishing the differences between the corpora lutea of menstruation and those of impregnation.

At page 96, we are informed that Professor Jackson is a decided advocate of the hypothesis, that the menstrual flow is a hemorrhage and not a secretion. The Editor might have added the name of his predecessor, and numerous others. Every microscopic observer now appears to favor that view of the subject.

At page 100, it is said gravely, that "a majority of the women, in the city of Philadelphia cease to menstruate at fifty years of age," and at page 205, that "the average length of children, born in Philadelphia, is from 18 to 22 inches." It may be so; but when, where, and how were the statistical evidences obtained?

Under fecundation we have a note of the ancient view of Professor Chapman, which referred every thing to sympathy; and that of Dr. Dewees, that the sperm is conveyed directly from the vagina to the ovaries "by a set of vessels not yet discovered:" both of these given without any comment. The latter gentleman, we believe, abandoned altogether his unsupported notion, and we apprehend that the former would scarcely wish the ashes of *his* to be disturbed.

At page 116, M. Velpeau states that the fundus of the uterus attains the level of the umbilicus, or even above it, at the end of the sixth month. On which the Editor makes the following note:—"The fundus does not rise as high as the umbilicus until the end of the *sixth* month of gestation."

Not exactly comprehending the meaning of the following note, we place it before the reader under the hope that he may be more fortunate:

"Most of the Lecturers on Midwifery, in this city, maintain that no relaxation of the ligamentous bands that surround the symphyses of the pelvis, take [takes] place during parturition, but that *the intervening cartilages are designed to prevent, by their elasticity, the effects of blows upon the bony girdle:* my own observations, however, lead me to unite with Velpeau on this subject."*

We are pleased to observe the allusion of the Editor to the merits of his "late highly talented pupil and friend"—[we are happy to say not dead, but likely to live a long life of usefulness]—Dr. E. K. Kane, in a note on Kiesteine as a "rational sign" of pregnancy: but our intelligent friend will blush at "the honor of establishing the unquestionable value of Kiesteine as a diagnostic sign of pregnancy" having been "reserved" for him. The value of his paper consisted essentially in the careful and protracted observation, and the unquestioned ability, and entirely trustworthy character of the Reporter; yet the results were not more decisive than those of Mr. Letheby, who observed the pellicle in 48 cases of pregnancy out of 50. We may add that a better attention on the part of the Editor to the excellent communication of his pupil, would have enabled him to make his note more in accordance with observed "*Sympathetic Phenomena.*"

What, we ask, can be the meaning of the following extract from a note on Superfœtation?—

"The modern doctrine of conception, termed *Epigenisis* [Epigenesis] is sustained, it is true, by many well-established facts, and by the highest medical authority, yet I confess that my confidence in its validity has been shaken by reading cases of superfœtation that have been recorded by medical gentlemen, although at variance with their preconceived hypotheses."—p. 218.

What possible connection can there be between Epigenesis and the cases of Superfœtation, referred to by the annotator? Epigenesis is the theory of conception, according to which the new being receives from each parent the materials necessary for its formation!!

The longest note relates to a case of sexual ambiguity, which is, in many respects, very far from satisfactory; and to the whole is appended a chapter on Puerperal Fever; the term *puerperal* being, the Editor thinks, "more comprehensive" than *childbed.* He is, indeed, as we have seen, by no means happy in his terms.

* The italics are our own.—Reviewer.

Under Puerperal Fever we are told of "a variety which we call *puerperal adynamia.*" We may ask, by the way, *en passant*, who Peauteau is, who wrote nearly a century ago, that puerperal fever is of the nature of erysipelas: we presume he means Pouteau. We find also Stall cited—a name altogether unknown to us as that of a writer on puerperal fever, or, indeed, on any thing else. "Had we space," the Editor remarks, "we could multiply authorities to show the intimate connection or simultaneous prevalence of these two diseases. Besides, we often observe, in other maladies, that erysipelas attacks the internal surface. In scarlatina maligna, it [quære, erysipelas?] assails the mucous lining of the throat, and not its dermoid covering."—p. 586. We confess we are at a loss to separate "*the mucous lining of the throat*" from its "*dermoid covering!*"

We believe we have cursorily noticed the most important notes of the Editor of the present edition. From them the reader will be enabled to judge whether the value of the work is materially enhanced by them, and whether, if the second edition had been reprinted without modification or additions, the loss to the student would have been calamitous.

As far as the mechanical execution of the volume is concerned, we have nothing to object. It is well got up. **

